# John C. Barker & Co.’s Cod Liver Oil

**Published:** 1885-10-20

**Authors:** 


### EDITORIALS AND MISCELLANEOUS.

JOHN C. BAKER & CO.'S COD LIVER OIL.—We regard this as a superior preparation of Cod Liver Oil. Its purity and value has stood the test of long experience. Its emulsion with Hypophosphites by this House is highly approved. See their advertising page of this journal.

